# p54-Fc-Labeled Gold Nanoparticle-Based Lateral Flow Strip-Assisted Portable Devices for Rapid and Quantitative Point-of-Care Detection of ASFV Antibodies

**DOI:** 10.3390/bios15010025

**Published:** 2025-01-06

**Authors:** Yang Yang, Yuhao Li, Ziyang Wang, Minglong Tong, Pengcheng Zhu, Juanxian Deng, Zongjie Li, Ke Liu, Beibei Li, Donghua Shao, Zhongren Zhou, Yafeng Qiu, Zhiyong Ma, Jianchao Wei

**Affiliations:** 1 Shanghai Veterinary Research Institute, Chinese Academy of Agricultural Sciences, Shanghai 200241, China; 15776623916@163.com (Y.Y.); liyuhaoaiwojia@163.com (Y.L.); wzy19991114@163.com (Z.W.); lizongjie@shvri.ac.cn (Z.L.); liuke@shvri.ac.cn (K.L.); lbb@shvri.ac.cn (B.L.); shaodonghua@shvri.ac.cn (D.S.); yafengq@shvri.ac.cn (Y.Q.); 2 Yixing Customs, Yixing 214200, China; caualong@163.com (M.T.); yixingzpc@139.com (P.Z.); 3 Nanjing Customs, Nanjing 210001, China; wangshm01@sina.com; 4 Shanghai Quicking Biotech Co., Shanghai 201314, China; info@quicking.cn

**Keywords:** immunochromatographic (IC) test, SPA, Fc fusion protein, ASFV, field rapid detection

## Abstract

In this study, a novel rapid immunochromatographic (IC) test for African swine fever virus (ASFV) antibodies is presented. An immunochromatographic test (IC) is a detection technique that combines membrane chromatography with immunolabeling. This approach saves time for antibody preparation, resulting in a shorter production cycle. p54 is an important structural protein of African swine fever, and an ideal protein for serotype diagnosis. Gold nanoparticles are attached to the ASFV p54-Fc fusion protein, and the ASFV-specific antigen p54 and Staphylococcus aureus protein A (SPA) are labeled on a nitrocellulose membrane, at positions T and C, respectively. We developed a SPA double sandwich IC test strip, and assessed its feasibility using ASFV p54 and p54-Fc fusion proteins as antigens. ASFV p54 and p54-Fc fusion proteins were expressed and purified. A sandwich cross-flow detection method for p54, which is the primary structural protein of ASFV, was established, using colloidal gold conjugation. Our method can detect ASFV antibodies in field serum samples in about 15 min using a portable colloidal gold detector, demonstrating high specificity and sensitivity (1:320), and the coincidence rate was 98% using a commercial ELISA kit. The dilution of the serum sample can be determined by substituting the absorbance (T-line) interpreted by portable devices into the calibration curve function formula of an African swine fever virus standard serum. In summary, our method is rapid, cost-effective, precise, and highly selective. Additionally, it introduces a new approach for constructing IC test strips using SPA protein without antibody preparation, making it a reliable on-site antibody test for ASFV.

## 1. Introduction

African swine fever (ASF), caused by the African swine fever virus (ASFV), is a highly lethal swine fever disease affecting young herds [1]. After its discovery in Kenya in 1921, the first case in China was reported in Shenyang in August 2018, causing enormous economic damage to the country’s stock farming industry [2,3]. Now, ASF is prevalent in several countries [4]. Unfortunately, no effective vaccine or treatment exists to control ASF outbreaks. The current control strategy of ASF relies mainly on the implementation of hygiene measures and the culling of infected and exposed animals [2].

ASFV is a complex icosahedral double-stranded DNA virus with a 200 nm diameter envelope, belonging to the Asfaviridae family, of the genus *Asfivirus* [5]. The ASFV genome spans about 170 to 190 kb, encoding over 151 proteins [6]. Virus particles have a five-layered structure, comprising the nucleoid, core shell, inner envelope, capsid, and outer envelope [7]. The p54 protein, encoded by the *E183L* gene, is a crucial structural component of ASFV, with a molecular weight of approximately 25 kDa, and contains a transmembrane domain [8]. p54 plays a vital part in viral morphogenesis and infection, facilitating the recruitment of virus particles in the endoplasmic reticulum during virus formation and post-assembly transfer [9,10]. p54 is also crucial in the initial stages of viral infection, inducing protective immune responses [11]. The levels of anti-p54 antibodies increase in swine infected with ASFV or inoculated with p54 replicons [12,13]. Anti-p54 antibodies become detectable in pigs around eight days after ASF infection, and persist in infected animals’ blood for several weeks [14,15]. The ASFV p54 protein has been used to establish many ELISA serological diagnostic methods, all of which have good application prospects [16,17,18]. Yanni Gao et al. developed a block ELISA assay based on p54, with high sensitivity, specificity, and stability [14]. Chaohua Huang et al. developed a blocking ELISA method, based on virus-like chimeric nanoparticles, with ASFV p54 epitopes for the serological diagnosis of ASFV [19]. The competitive ELISA, based on a p54 monoclonal antibody, developed by Weldu Tesfagaber et al. displayed high diagnostic sensitivity and specificity for ASFV antibody detection [10]. Chaohua Huang et al. developed a closed ELISA, based on p54 chimeric BTV CLP, which provides a new tool for ASFV monitoring [19]. Therefore, the p54 protein is an ideal ASFV antigen for serological diagnostic tests.

At present, culling and strict hygiene procedures are priority measures to control African swine fever in domestic pigs. The detection of ASFV antibodies, which indicate ASFV infection, is key to disease prevention and control [20]. Thus, it is significant to conduct highly sensitive and specific diagnostic analysis in order to realize the rapid and early detection of infected pigs. Serological diagnosis tests are commonly used for their simplicity, cost-effectiveness, and safety, without the need to isolate the virus or extract its genome [21].

Staphylococcus aureus protein A (SPA) is a surface protein with 4–5 homologous immunoglobulin binding domain residues, a molecular weight of 40–60 kDa, a polymorphic variable repeat region Xr and a conserved region Xc, which is isolated from the cell wall of bacteria [22]. SPA is a well-known Gram-positive bacterial cell wall protein that binds to the Fc region of various mammalian IgG antibodies, and is suitable for radioimmunoassays and enzyme-linked immunosorbent assays (ELISA) [23]. SPA effectively binds to the Fc region of nearly all human antibodies, except IgG3, as well as specific animal antibodies, like those of pigs, dogs, rabbits, goats, and mice [24,25]. SPA plays a major role in various biochemical, biotechnological, and medical applications. In immunochromatographic (IC) test strips, SPA is used for its binding capacity with mammalian serum IgG molecules, presenting antibodies for detection by reacting with labeled antigens on the strip [24].

Rapid, sensitive, and field-deployable ASFV tests are vital for ASF surveillance and control. Current early ASF diagnosis methods include immunoblotting tests (IBT), immunofluorescence assays (IFA), enzyme-linked immunosorbent assays (ELISA), colloidal gold IC assays, polymerase chain reactions (PCR), TaqMan^®^ PCR assays, fluorescence quantitative PCR, cross-initiated amplification (CPA) assays, polymerase cross-linking spirogram reaction (PCLSR) analysis, etc. [26]. While these methods offer high sensitivity and accuracy, some are time-consuming, require costly equipment, and demand specialized personnel, limiting their field utility. PCR, real-time PCR, and ELISA are recommended by the OIE (World Organization for Animal Health), but ASFV attenuation variants pose challenges for nucleic acid-based diagnostics (Table 1). Therefore, a highly effective serological test for detecting ASFV antibodies is crucial for ASF prevention and control. The colloidal gold IC test strip method offers advantages, such as ease of use, practicality, speed, low cost, portability, high specificity, and the ability to be interpreted by the naked eye [27,28]. It is suitable for rapid field diagnosis of ASFV, epidemiological investigations, and monitoring of acute and suspected infections in pigs [29]. Therefore, the colloidal gold IC test strip assay was chosen for this study.

## 2. Materials and Methods

### 2.1. Serum Samples

A total of 10 inactivated positive sera of ASFV were infected, and 10 negative sera were acquired from the China Animal Health and Epidemiology Center, Qingdao, China. In addition, a total of 100 samples of standard ASFV positive and negative inactivated sera from the China Animal Health and Epidemiology Center, Qingdao, China were used for ROC curve analysis. All positive sera were tested using the commercial ASFV antibody ELISA kits (Beijing Jinnuo Baitai Biotechnology Co., Ltd., Beijing, China (JN60915) (based on p30 protein)). A total of 100 inactivated clinical serum samples were collected from pig farms suspected of having ASFV infections in eastern China in 2023 to calculate the coincidence rate, with the commercially available ASFV antibody ELISA kit (Beijing Jinno Batai Biotechnology Co., Ltd. (JN60915), based on the p30 protein). The Japanese encephalitis virus (JEV), classical swine fever virus (CSFV), porcine circovirus type 2 (PCV2), pseudorabies virus (PRV), Getah virus (GETV), and porcine reproductive and respiratory syndrome virus (PRRSV) from experimentally infected pigs, supplied by the China Animal Health and Epidemiology Center (Shanghai Branch), were used to realize specific detection.

### 2.2. Production and Identification of Recombinant African Swine Fever Virus p54 and p54-Fc Proteins

The *p54* gene of ASFV SY-18 (GenBank entry number MH766894.1) and the *Fc* gene of porcine IgG were codon-optimized according to the prokaryotic expression system, and synthesized by Tsingke Biotechnology, China. The p54-Fc fusion gene was amplified by fusion PCR, using the primers listed in Table 2.

PCR products of the *p54* and *p54-Fc* genes were ligated into the pCold I vector to construct respective recombinant expression plasmids. The constructed p54 and p54-Fc plasmids were transformed into *E. coli* BL21 cells and induced with IPTG for the expression of p54 and p54-Fc recombinant proteins. The recombinant p54 and p54-Fc proteins were purified by Ni+ affinity chromatography and detected by SDS-polyacrylamide gel electrophoresis (SDS-PAGE) and Western blot (WB), using anti-His (dilution 1:5000) and ASFV-positive serum as primary antibodies. An HRP-conjugated protein A (BOSTER Biological Technology, Wuhan, China) was used to detect p54 and p54-Fc proteins by WB.

### 2.3. Preparation of p54-Fc Protein with Colloidal Gold Conjugate

The desired glass containers were acidified, silicified, and then dried after rinsing with H_2_O ten times. The colloidal gold was prepared by the reduction method, using trisodium citrate, and stored at 4 °C in the dark. The size and morphology of the prepared colloidal gold and the dispersion of the gold particles were observed under transmission electron microscopy.

### 2.4. Preparation of IC Test Strips and Test Results

The test strip consisted of a PVC-backed card sample pad, a conjugate pad, an absorption pad, and a nitrocellulose (NC) membrane. The conjugation pad was prepared when it was spread with the p54-Fc labeled with an optimum concentration of colloidal gold. The control line was sprayed with a standard SPA protein (BOSTER Biological Technology, China). The sample pad, coupling pad, NC membrane and absorption pad were successively attached to the PVC backing card, overlapping by 1–2 mm. Finally, the cards were cut into strips, 2.9 mm × 60 mm wide, and assembled into a plastic shell.

**Figure 1:**
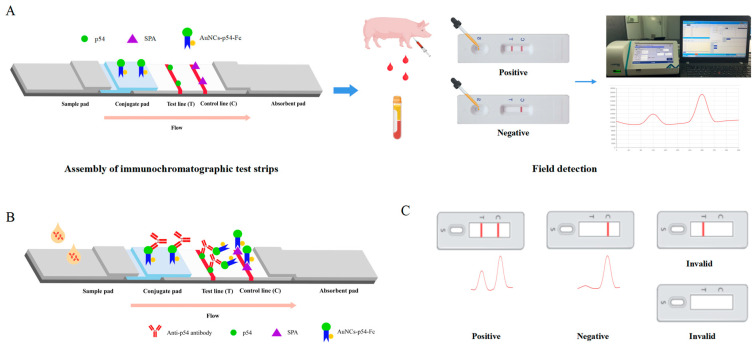
Design of an immunochromatographic test strip for the detection of ASFV antibodies. (**A**) Design process and (**B**) schematic of the test strip for ASFV antibody detection. (**C**) Positive and negative results were detected by the test strip.

The assembly of an IC test strip is shown in Figure 1. In the presence of specific anti-p54 antibodies in the serum, they were recognized by colloidal gold labeled p54-Fc to form antibody–antigen immune complexes. Then, these complexes were captured by the p54 protein labeled on the test line (T) to form a double antigen sandwich, generating a red band that was visible to the naked eye. In the absence of anti-p54 antibodies, there was no display of a red band on the test line (Figure 1B). For a valid test, the SPA protein can capture colloidal gold-labeled p54-Fc as a visible red band at the control line (C), irrespective of the presence or absence of anti-p54 antibodies in the serum. In the absence of a red strip on line C, the test was considered invalid (Figure 1B,C). The strips at the end of the reaction were detected using a portable colloidal gold immunochromatography analyzer (LABIM, Shanghai, China). After the detection began, the instrument quickly displayed the detection values of T and C lines. According to the reflection spectrum test method, the absorption and reflection of colloidal gold particles on light at a specific wavelength is related to the amount of colloidal gold particles, and the light absorption and reflection degree on the T line and C line of colloidal gold card was detected by the photoelectric sensor to reflect the residual amount of colloidal gold particles (Figure 1A,C). The darker the color of the T and C lines of the strip observed by the naked eye, the more colloidal gold particles remained, and therefore the higher the value detected by the instrument.

In order to analyze the performance of IC test paper for ASFV detection, a total of 100 ASFV positive and negative standard sera were analyzed with the IC test strips. After the IC test strips reacted for 15 min, the fluorescence intensity was read using a portable card reader. Based on the fluorescence intensity value, SPSS 26.0 software was used to analyze the ROC curve to evaluate its performance [34].

### 2.5. Quantitative Analysis Using IC Test Strips

To quantify the amount of ASFV antibody, a calibration curve is measured by testing a standard positive serum. This calibration curve provides a quantitative relationship between ASFV antibody dilution and absorbance (T-line). The number of ASFV antibodies can be quantified according to the standard curve drawn. ASFV standard antibody solutions were prepared using ASFV antibody-positive standard sera in dilution buffer at final dilutions of 1:10, 1:20, 1:40, 1:80, 1:160, 1:320, 1:640.

### 2.6. Determination of Sensitivity and Specificity of IC Strips

ASFV-positive sera were diluted at 1:20, 1:40, 1:80, 1:160, 1:20 20, 1:640, and 1:1280 to determine the sensitivity of the test strips. ASFV-, CSFV-, PRV-, PRRSV-, PCV2-, GETV-, and JEV-positive serum samples were tested to evaluate the specificity of the test strips.

### 2.7. Reproducibility and Stability of IC Test Strips

The reproducibility of three different IC test strips coated in the same batch were tested by using the established IC test strip detection method in six serum samples. Then, three different batches of coated IC test paper were selected for a repeatability test at random. The intra-assay and inter-assay variability [coefficient of variation (CV)] of each group was calculated.

The prepared test strips were sealed, dried, and stored at 37 °C for 30 days. The test strip was stored at 37 °C for 30 days, which is equivalent to half a year at room temperature. Ten ASFV-positive and ten ASFV-negative serum samples with known background information were tested with the stored strips to determine their stability.

### 2.8. IC Test Strips and Commercial ELISA Kit

This study compared the results of IC test strips with those of the ASFV ELISA antibody test kit, using 100 clinical samples. The 100 clinical sera were detected by using the IC strip and ASFV ELISA kit established in this study, respectively. The experimental results of the two methods were compared and the coincidence rate was calculated, and the method to calculate the coincidence rate was the following: consistency rate = [(true positive + true negative)/(true positive + true negative + false negative + false positive) × 100%] [35].

## 3. Results

### 3.1. Expression and Purification of Recombinant p54 and p54-Fc Proteins

**Figure 2:**
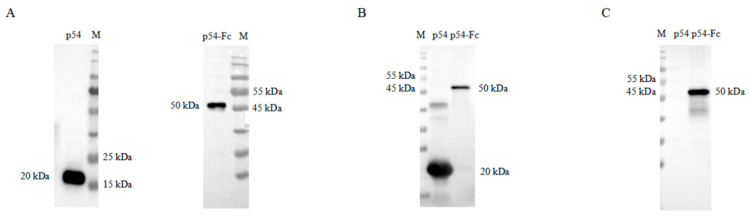
ASFV p54 and p54-Fc protein expression. (**A**) The expression of p54 and p54-Fc proteins was detected by anti-His monoclonal antibodies. (**B**) p54 and p54-Fc protein expressions were detected in porcine-positive serum. (**C**) p54-Fc protein expression was detected by HRP-conjugated protein A.

In this study, p54 protein and P54-FC fusion fragment of ASFV were expressed using a pCold expression vector. Recombinant p54 and p54-Fc fusion proteins were identified by anti-His and ASFV-positive serum antibodies by WB, indicating their good antigenicity (Figure 2A,B). HRP-conjugated protein A was used to detect p54 and p54-Fc proteins by WB. While p54-Fc protein showed target bands, p54 protein showed no bands (Figure 2C). These results indicated the SPA-binding ability of the p54-Fc protein.

### 3.2. Preparation and Condition Optimization of Colloidal Gold Conjugate p54-Fc Protein

**Figure 3:**
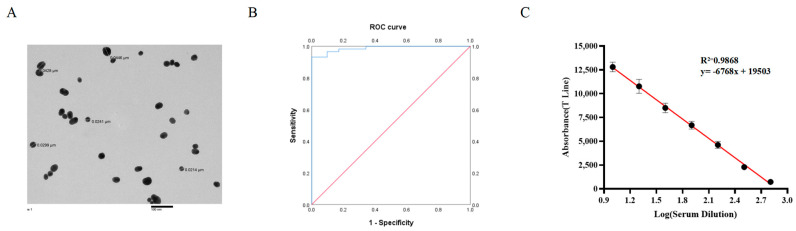
Preparation and condition optimization of colloidal gold-conjugated p54-Fc protein. (**A**) Transmission electron microscope image of gold nanoparticles (AuNPs). (**B**) Receiver operating characteristic (ROC) analysis of the developed immunochromatographic test strip. The blue line represents the test curve, and the red line corresponds to the noninformative test curve. The sensitivity and specificity of the IC test strips were 93.2% and 97.6%, respectively, when the optimal cut-off absorbance value was 977.84. The area under the ROC curve (AUC) of the IC test strips was 0.988 (97.3% CI, 0.973−1.000). (**C**) Fitting the linear relationship between the Absorbance (T line) and dilutions.

To enhance the function of the IC strip test, a preliminary test of the amount of p54-Fc protein labeling was investigated. The optimal protein labeling amounts were 40 μg/mL. The grain size, morphology, and dispersion of the wine-red gold particles prepared with sodium citrate were observed by transmission electron microscopy. As shown in Figure 3A, the prepared gold particles were relatively uniform and dispersed, with a particle size of about 30 nm.

### 3.3. ROC Curve Analysis

After analyzing the 100 pig serum samples collected with ELISA, this study made a comparison between the results of this analysis with the IC test strip. With the use of SPSS 26.0 software, ROC analysis indicated that the area under the curve (AUC) of the IC test paper was 0.988 (95% confidence interval [CI], 0.973−1.000; Figure 3B), suggesting that the IC test strips were effective in differentiating between positive and negative samples. According to the Youden’ s index of the ROC curve, the critical value is determined to be 977.84 [34]. Therefore, the IC test strip constructed in this study was judged as positive if the detection value was higher than the critical value of 977.84, and negative if the detection value was lower than the critical value of 977.84. Equal to the critical value, 977.84 was judged suspicious. The suspicious samples were retested, and were judged negative for samples less than 977.84 and positive for samples greater than 977.84.

### 3.4. Linearity of Absorbance (T-Line) Versus Dilution of African Swine Fever Virus Standard Serum

The absorbance (T-line) of different dilutions of standard positive serum (1:10–1:640) was plotted as a calibration curve. As can be seen in Figure 3C, the calibration curve is well linear in the range of 1:10–1:640 with a reliable correlation coefficient (R^2^ = 0.9868).

### 3.5. Sensitivity of IC Test Strips

**Figure 4:**
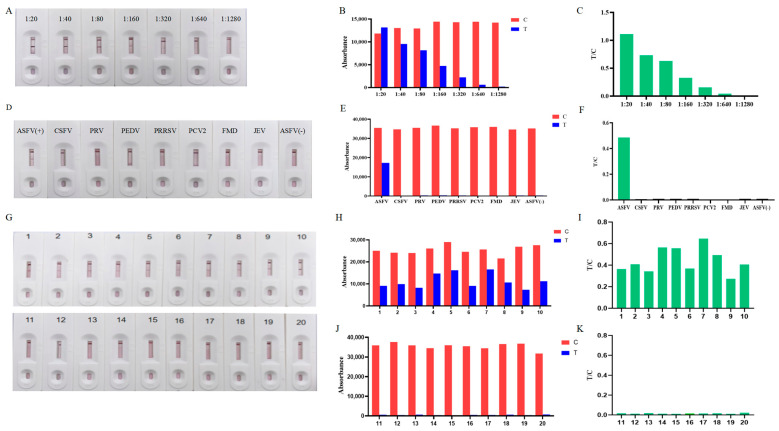
Sensitivity and specificity of the p54-Fc immunochromatographic test strip. (**A**) The sensitivity of the test strip was detected against various dilutions of ASFV-positive serum from 1:20 to 1:1280; the highest dilution detected by this method was 1:320. (**D**) Specificity of the test strip was detected against ASFV-positive and negative serum, PRRSV, JEV, GETV, CSFV, PCV2, PPV, and PRV antibodies positive porcine serum. Except for the ASFV-positive serum, there were no bands on the T-line of other test serum strips. (**G**) Stability of the test strip. (**B**,**E**,**H**,**J**) The absorbance values of the test line (T-line) and control line (C-line) of the lateral flow assay. (**C**,**F**,**I**,**K**) The T/C value of the lateral flow assay.

The ASFV-positive serum was diluted at 1:20, 1:40, 1:80, 1:160, 1:320, 1:640, and 1:1280 to check the sensitivity of the IC test strips. The absorbance of the T-line linearly decreased with the increasing dilution of the antibodies (Figure 4A, from left to right). The test strip at the end of the reaction was put into the colloidal gold detection instrument, and the detection values were shown in Figure 4B,C. For the 1:320 serum, the T-line band can be seen on the colloid gold test strip, and the detection value was positive. For the 1:640 serum, the T-line band became very thin and almost invisible to the naked eye, and the test value is negative (Figure 4B,C). The darker the color of the strip observed by the naked eye, the higher the detection value. Therefore, we found the sensitivity of this detection method to be 1:320.

### 3.6. Specificity of IC Test Strips

To evaluate the specificity of the IC test strip, we detected ASFV-, CSFV-, PRV-, PEDV-, PRRSV-, PCV2-, FMD-, and JEV-positive serum samples. As shown in Figure 4D, the test strip C-line indicated a valid test. However, only the ASFV-positive serum samples produced clear red bands on the T-line, while the other samples failed to do so. The test strip was detected using a colloidal gold detection instrument, and the test values were shown in Figure 4E,F. This indicated the high specificity of the IC test strip (Figure 4D–F). The IC test strip used in this test used high purity specific proteins to capture antibodies in serum, so it has a high specificity.

### 3.7. Repeatability and Stability of IC Test Strips

In order to verify the repeatability of the IC test strip method, the intra-determination variation and inter-determination variation were calculated. The maximum CV of the intra-assay repeatability test was 4.05% (Table 3), and the maximum CV of the inter-assay repeatability test was 6.56% (Table 3), both <10%. These results indicate that the established IC test strips method has good repeatability.

To test the stability of IC test strips, we first stored them at 37 °C for 30 days, and then tested 10 ASFV positive and 10 ASFV negative sera. The details are mentioned in Section 2. The results showed that all 10 ASFV-positive sera showed two bands (C-line and T-line), while ASFV-negative sera showed only one C-line. This indicated that the IC test strips were stable at 37 °C for 30 days, indicating their good stability (Figure 4G–K).

### 3.8. Comparison of IC Test Strips with a Commercial ELISA Kit

A total of 100 clinical samples were tested using the IC test strips and the commercial ASFV antibody ELISA kits (Beijing Jinnuo Baitai Biotechnology Co., Ltd., (JN60915), based on a p30 protein. We compared the detection results of the two detection methods and calculated the coincidence rate. The results showed that a total of 31 positive and 69 negative ASFV samples were detected using the commercial ASFV antibody ELISA kit. A total of 32 positive and 68 negative ASFV were detected using IC test strips. The coincidence rate between the IC test strip and the ASFV ELISA antibody kit was 98%, indicating excellent reproducibility (Table 4).

## 4. Discussion

ASF has caused substantial economic losses to the Chinese pig industry. The international community has yet to develop a secure and effective vaccine or treatment for ASF. Thus, early detection and monitoring remain essential for controlling the spread of AFSV [37,38]. Clinical diagnosis of ASF requires methods, such as virus isolation, immunofluorescence, serological and nucleic acid detection, and immunohistochemistry. The serological approach is widely used for its simplicity, cost, effectiveness, and precision [14].

Therefore, to control and eradicate ASF, it is necessary to screen specific and sensitive antigen proteins and establish a high-sensitivity and specificity serological assay for ASFV. Among the currently commonly used serological diagnostic methods, the colloidal gold IC strip does not require special equipment or professional knowledge. The test is simple and convenient to operate, and the results can be quickly detected by the naked eye within 10 min [29]. At the same time, the test strip can also be used to scan the reading of the instrument for quantitative detection. In addition, two new monoclonal antibodies recognizing different epitopes of p30 have been developed [21]. These can be used to produce effective IC test strips for ASFV detection. The researchers used a monoclonal antibody against VP72, which is the main viral capsid protein of ASFV, to establish an IC viral antigen detection test [5]. p30, p54, and p72 are the established serological diagnostic proteins among the more than 150 proteins expressed by ASFV. p54, a membrane protein, is expressed in the early stages of ASFV infection. It has good antigenicity and immunogenicity, and is relatively conserved [39]. Therefore, p54 can be used for the serological diagnosis of ASFV [40].

In the absence of ASFV vaccination, the presence of ASFV antibodies indicates a history of infection. Therefore, antibody testing is particularly important. Currently, there are multiple ASFV virulence gene deletions resulting in weakened, long-lasting infection strains as well as latent infections [41]. Additionally, antibodies from ASFV-infected or recovered pigs can remain in their bodies for a long time. Therefore, antibody-based surveillance can help detect surviving pigs, elucidating the epidemiological characteristics of ASFV. Moreover, the establishment of antibody detection methods is also critical for disease eradication programs [42].

In this study, a novel IC test strip was developed for detecting ASFV using ASFV p54 and SPA proteins. This IC test strip exploits SPA’s ability to bind to the Fc segment of IgG. The innovation in this method lies in the fact that the p54-Fc fusion protein exhibits strong binding capability with the SPA protein, which serves as the quality control line. The p54 protein functions as a test line for detecting p54 antibodies in serum (Figure 1A). This study not only established an ASFV antibody detection method, but also explored a platform for constructing colloidal gold IC test strips. The ASFV p54 protein on the test line can be substituted with the major antigenic protein of any virus, and the colloidal gold-labeled p54-Fc fusion protein on the conjugate pad can be replaced with the corresponding Fc fusion protein, with SPA protein consistently occupying the control line (Figure 1). The detection complex formed by the previous immunochromatographic detection method for the diagnosis of African swine fever serum was composed of gold nanoparticle–antibody–antigen at line C [27], while the complex formed by the immunochromatographic test strip detection method in this study was SPA-p54-Fc complex at line C. Therefore, this method allows the detection of ASFV antibodies in serum without requiring antibody preparation, simplifies the test procedure, reduces the time and test cost, and minimizes non-specific antibodies. SPA protein, a readily available commercialized protein, rapidly binds to Fc. Our IC test has strong specificity, repeatability, and stability, with a sensitivity reaching 1:320 and a storage period of 30 days at 37 °C. Its coincidence rate with the ASFV-blocking ELISA kit is 98%.The method constructed in this experiment also had the potential for quantitative detection with R^2^ = 0.9868 (Figure 3C). The antibody detection technology is simple to operate, has short field portable detection time, has high sensitivity and specificity detection capabilities, and may be applied to the field detection of ASFV in pig farms as a potential tool.

While ELISA offers similar assay sensitivity and can be integrated into automated procedures, it demands laboratories, skilled technicians, and specialized equipment, with the entire process taking about 4 h. Consequently, on-site testing is not feasible using ELISA (Table 1). In contrast, our IC test is advantageous as follows: (1) it requires no specialized knowledge or equipment, making it straightforward to operate; (2) it provides faster results within 15 min of observation time; (3) it can quantitatively detect the ASFV antibody content in serum according to the standard curve drawn; and (4) it is more cost-effective, making it suitable for use in primary health care units and on-site testing.

P Sastre et al. have developed an ASFV VP72-based lateral flow analysis (LFA) for early detection of ASFV infection, and for use in small laboratories with very simple or lacking laboratory equipment [5]. Using treatment of p72 with acid labeled with gold nanoparticles, Zhu Wenzhuang et al. developed a side-flow chromatography method for the detection of ASFV antibodies that combines the advantages of an immune side-flow system with the unique properties of gold nanoparticles [27]. Rui Geng et al. developed a p72 trimer-based colloidal gold immunochromatographic assay strip that can be used as a complement to nucleic acid detection, and this band can accurately identify ASFV antibodies in pig serum [29]. Notably, side-flow strips outperform commercial ELISA kits in terms of cost effectiveness, stability, reaction time, and ease of use. To date, side-flow strips based on double quantum dot (QDM) microspheres, latex microspheres, or fluorescent microspheres have also been developed for ASFV antibody detection [43,44,45]. Compared with these methods mentioned above, the gold nanoparticle-based test strip developed in this study owns obvious advantages, as listed in Table 1: simple operation, no expensive equipment, shorter time and intuitive results.

In summary, a novel concept was employed to create antibody detection strips by combining an Fc protein and SPA. ASFV serum antibodies can be detected without the need for prior antibody preparation. This method exhibits a certain degree of versatility and can be used for constructing surface chromatography test strips. Our IC test strip holds potential application value in the surveillance and control of ASFV outbreaks.

## Figures and Tables

**Table 1:** Comparison of ASFV serological detection methods.

Method	Application Scenario	Time	Sensitivity	Specificity	Cost	Advantages	Disadvantage	Reference
ELISA	Laboratory testing	>2 h	1:256– 1:6400	High	USD 8 per sample	High sensitivity	Requirement for a diagnostic device	[30]
FAT	Laboratory testing	>2 h	1:400	High	USD 20 per sample	Easy to operate	Non-specific interference, Expensive equipment	[31]
Immunocytochemistry	Laboratory testing	>2 h	1:400	High	>USD 20 per sample	High specificity and sensitivity	Time-consuming, Technical complexity	[32]
CLIA	On-site testing	1 h	1:128	low	>USD 20 per sample	High sensitivity, Results stable	False positive, High cost	[33]
Colloidal gold test strip	On-site testing	15 min	1:320	High	USD 2 per sample	Simple operation, short detection time, Intuitive results	Low sensitivity	This study

**Table 2:** Primers used for polymerase chain reaction assay.

Primer Name	Primer Sequence (5′-3′)
p54-F	GGAATTCATGGATTCTGAATTTTTTCA
p54-R	GCTCTAGATTACAAGGAGTTTTCTAGGTC
Fc-F	GCGGAGGTGGCTCTGGCGGTGGCGGATCGATCTGTCCGGCATGTG
Fc-R	GCTCTAGATTTACCCGGGGTTTTG

**Table 3:** The results of the repeating test.

Sample Number	The Result of Intra			The Result of Inter		
	Average Value	Standard Deviation	Coefficient Variation	Average Value	Standard Deviation	Coefficient Variation
1	6296.61	248.01	3.94%	13,596.61	419.17	3.08%
2	7219.57	131.16	1.82%	9219.57	131.12	1.42%
3	5647.34	228.56	4.05%	11,980.68	785.81	6.56%
4	11,249.01	115.18	1.02%	6315.68	132.29	2.09%
5	16,275.76	511.07	3.14%	10,942.43	480.41	4.39%
6	17,352.34	505.31	2.91%	7531.67	447.78	5.95%

**Table 4:** Comparison of an immunochromatographic test strip with a commercial ELISA kit.

Detection Method		Commercial ASFV ELISA			
		Positive	Negative	Total	Kappa Statistic
Immunochromatographic test strip	Positive	31	1	32	0.954
	Negative	1	67	68	
	Total	32	68	100	
Coincidence rate		96.88%	98.52%	98%	

Relative sensitivity = 31/32 = 96.88%, relative specificity = 67/68 = 98.52%, overall coincidence rate = 98/100 = 98%. Relative sensitivity is also called true positive rate: the higher the sensitivity, the lower the missed diagnosis rate. Relative specificity is also called true negative rate: the higher the specificity, the lower the misdiagnosis rate. Kappa statistics are used in statistics to assess the consistency of classification or measurement methods. A kappa value of 0.0~0.20 indicates very low consistency, 0.21~0.40 general consistency, 0.41~0.60 medium consistency, 0.61~0.80 high consistency, and 0.81~1 almost complete consistency [36].

## Data Availability

No new data were created or analyzed in this study. Data sharing is not applicable to this article.
